# Circ_0020093 ameliorates IL-1β-induced apoptosis and extracellular matrix degradation of human chondrocytes by upregulating SPRY1 via targeting miR-23b

**DOI:** 10.1007/s11010-021-04186-2

**Published:** 2021-05-27

**Authors:** Mingli Feng, Lin Jing, Jingbo Cheng, Shuai An, Jiang Huang, Qi Yan

**Affiliations:** 1 Department of Orthopaedics, Xuanwu Hospital, Capital Medical University, Changchun Ave 45, Xicheng District, Beijing, 100053 China; 2 Department of Orthopaedics, Wangjing Hospital, China Academy of Chinese Medical Sciences, Beijing, China

**Keywords:** circ_0020093, miR-23b, SPRY1, Chondrocyte, Osteoarthritis, IL-1β

## Abstract

Osteoarthritis (OA) is a chronic disease characterized by articular cartilage degeneration and uncontrolled chondrocyte apoptosis. At present, accumulating evidence introduces that circular RNA (circRNA) is involved in the development of OA. The aim of our study was to explore the role and the functional mechanism of circ_0020093 in OA cell model. Human chondrocytes were treated with interleukin-1 beta (IL-1β) to construct OA model. The expression of circ_0020093, miR-23b, and Sprouty 1 (SPRY1) mRNA was measured by quantitative real-time polymerase chain reaction (qRT-PCR). Cell apoptosis was assessed by flow cytometry assay. The expression of extracellular matrix (ECM)-associated markers and SPRY1 protein level was detected by qRT-PCR and Western blot. Bioinformatics analysis-predicted relationship between miR-23b and circ_0020093 or SPRY1 was further verified by dual-luciferase reporter assay and RNA pull-down assay. In this study, we found that the expression of circ_0020093 and SPRY1 was declined, while miR-23b expression was elevated in IL-1β-treated chondrocytes. IL-1β induced chondrocyte apoptosis and ECM degradation, while these negative effects were alleviated by circ_0020093 overexpression or miR-23b inhibition. MiR-23b was a target of circ_0020093, and SPRY1 was a downstream target of miR-23b. Rescue experiments showed that miR-23b enrichment reversed the role of circ_0020093 overexpression, and SPRY1 knockdown also reversed the effects of miR-23b inhibition. Importantly, circ_0020093 positively regulated SPRY1 expression by targeting miR-23b. In conclusion, circ_0020093 ameliorates IL-1β-induced apoptosis and ECM degradation of human chondrocytes by regulating the miR-23b/SPRY1 axis.

**Supplementary Information:**

The online version contains supplementary material available at 10.1007/s11010-021-04186-2.

## Introduction

Osteoarthritis (OA) is the most common disease of synovial joints, characterized by cellular stress and extracellular matrix (ECM) degradation due to micro- and macro-damages, thereby activating maladaptive repair responses [1, 2]. It is estimated that by 2050, 130 million people worldwide will suffer from OA, and 40 million people will be severely disabled, highlighting the huge burden of this severe disease [3]. Recent evidence indicates that OA is a heterogeneous and multifaceted disease with various molecular and clinical phenotypes [4]. The survival of chondrocytes is essential for maintaining proper articular cartilage [5]. The study of the dynamic balance process of chondrocyte stability will help to understand the pathogenesis of OA and provide effective strategies for the treatment of OA.

The landscapes of circular RNA (circRNA) arouse many public interests because increasing evidence supports that circRNA is implicated in the development of numerous human diseases [6]. CircRNA is produced by precursor mRNA via “back-splicing” and identified by its unique closed-loop structure [6]. In OA, partial circRNAs have been presented to be deregulated in OA synovial fluid or in vitro chondrocyte models, and their functions have been preliminarily discussed. For example, Zhou et al. utilized interleukin-1 beta (IL-1β) to treat mouse chondrocytes, and circRNA expression profile showed that 119 circRNAs were strikingly upregulated, while 136 circRNAs were downregulated in IL-1β-treated chondrocytes compared to the untreated normal controls [7]. Wu et al. found that circ_0005105 was highly expressed in IL-1β-treated chondrocytes, and its reintroduction aggravated chondrocyte ECM degradation [8]. Another study performed RNAseq analysis to obtain differently expressed circRNAs in clinical OA tissues and controls, and circ_0020093 was proved to be deficient in OA tissues [9], which attracted our attention. Circ_0020093 is derived from attractin like 1 (ATRNL1), and its aberrant downregulation in OA implies that circ_0020093 may play a vital part in OA development.

Referring to the functional mechanism, endogenous circRNA is well known to work as microRNA (miRNA) molecular sponge, meaning that circRNA has the capacity to bind to miRNA and thus blocking miRNA expression [10]. The development of bioinformatics facilitates the screening of potential targets of circRNA [11]. Based on this, miR-23b was predicted to be a target of circ_0020093. A previous study claimed that miR-23b expression was promoted by tumor necrosis factor-α (TNF-α) stimulation in chondrocytes [12], hinting that miR-23b was also involved in OA development. However, the interplays between miR-23b and circ_0020093 in OA are not explored.

Sprouty 1 (SPRY1), a member of the SPRY gene family, plays wide biological functions in tumor initiation and progression, such as human epithelial ovarian cancer [13]. Besides, SPRY1 was also highlighted to be necessary to maintain the development of human chondrocytes [14]. However, the role of SPRY1 action in OA is not fully elucidated, and it is unknown yet whether SPRY1 is involved in the circ_0020093/miR-23b pathway.

In this study, we constructed OA model by treating human chondrocytes with IL-1β and monitored the expression pattern of circ_0020093 in IL-1β-treated chondrocytes. The function of circ_0020093 on cell apoptosis and ECM degradation was investigated, and the target relationship between miR-23b and circ_0020093 or SPRY1 was validated to establish a potential regulatory pathway. The objective of this study was to propose circ_0020093 as a promising biomarker for OA treatment.

## Materials and methods

### Cells, reagents and cell treatment

Human chondrocytes purchased from EK-Bioscience (Shanghai, China) were cultured in DMEM (EK-Bioscience) medium supplemented with 10% FBS (EK-Bioscience). Recombinant human IL-1β purchased from Absin (Shanghai, China) was dissolved in sterile distilled water (1.0 mg/mL) and preserved at − 20 °C for use.

Human chondrocytes were treated with IL-1β solution of 10 ng/mL concentration for different time (0, 12, 24 and 48 h), and untreated cells were used as the control.

### Cell transfection

For circ_0020093 overexpression, circ_0020093 sequence was cloned into pCD5-ciR vector (Geneseed, Guangzhou, China), naming as circ_0020093, with pCD5-ciR vector as a comparison. Small interference RNA targeting circ_0020093 or SPRY1 (si-circ_0020093 or si-SPRY1) and matched control (si-con) were synthesized by Ribobio (Guangzhou, China). The inhibitors and mimics of miR-23b (miR-23b and in-miR-23b), as well as matched controls (miR-con and in-miR-con) were obtained from Ribobio.

These plasmids and oligonucleotides were transfected into chondrocytes alone or together using Lipofectamine 3000 reagent (Invitrogen, Carlsbad, CA, USA) following the statement from protocol.

### Quantitative real-time polymerase chain reaction (qRT-PCR)

Total RNA from chondrocytes was extracted using Trizol reagent (Absin). The A260/280 value of isolated RNA was distributed from 1.8 to 2.0. Then, reverse transcription for circRNA and mRNA was performed using the First Strand cDNA Synthesis kit (TaKaRa, Dalian, China), and the amplification of cDNA was carried out using the SYBR Master Mix (Takara), with β-actin as the internal reference. Reverse transcription for miRNA was performed using the TaqMan MicroRNA Reverse Transcription Kit (Applied Biosystems, Foster City, CA, USA), and qRT-PCR amplification was performed using SYBR Master Mix (Takara), with U6 as the internal reference. The relative expression was calculated using the 2^−ΔΔCT^ method. The primer sequences are listed in Table 1.

**Table 1:** Primer sequences used in qRT-PCR

Name	Primer sequence (5′–3′)
circ_0020093	F: TTGCTCTGGTCATGGGAAGT
	R: TCTGTTAACCTTGCCAACTATCA
miR-23b	F: GCCCATTAGGGACCGTTA
	R: CTCAACTGGTGTCGTGGA
Aggrecan	F: AGGCTGAAGTTACAGGTC
	R: TTGGCTCCCAGTGTCTTA
COL2	F: ACTGGTAAGTGGGGCAAGAC
	R: CCACACCAAATTCCTGTTCA
MMP13	F: AGTTCGGCCACTCCTTAGGT
	R: TGGTAATGGCATCAAGGGAT
MMP3	F: CAAGGGCCTCTTCTGCGATTTCG
	R: CGGTAGGCAGCTAGGGCAGGGC
ADAMTS4	F: GATCGATGCCGGTGCTAAGA
	R: TCCTATGGGAGAACGGCAGA
SPRY1	F: CCCTGCCCTGGATAAGGAAC
	R: GGCCGAAATGCCTAATGCAA
β-actin	F: TGGACTTCGAGCAAGAGATG
	R: GAAGGAAGGCTGGAAGAGTG
U6	F: GCTTCGGCAGCACATATACTAA AAT
	R: CGCTTCACGAATTTGCGTGTCAT

*COL2* collagen II; *MMP13* matrix metallopeptidase 13; *MMP3* matrix metallopeptidase 3; *ADAMTS4* a disintegrin and metalloproteinase with thrombospondin motifs 4; *SPRY1* sprouty 1

### Flow cytometry assay

Cell apoptosis was examined using the Annexin V-FITC apoptosis assay Kit (Absin). In brief, cells were collected and exposed to Tyrisin. Then, cells were washed with PBS and resuspended in 300 μL binding buffer. A total of 5 μL Annexin V-FITC was added to label cells at room temperature for 15 min in the dark. Subsequently, 5 μL propidium iodide (PI) was added to incubate cells for another 5 min in the dark. The apoptotic cells were analyzed by a flow cytometer (BD Biosciences, San Jose, CA, USA).

### Western blot

Total protein was extracted using the Tractor Buffer Kit (Takara), and the concentration of protein was examined using the BCA protein assay Kit (Takara). Then, equal amount of protein was isolated by 12% SDS-PAGE and transferred into PVDF membrane (Absin). The membranes containing proteins were blocked with 5% non-fat milk for 1 h and then probed with the primary antibodies against Aggrecan (ab36861; Abcam, Cambridge, MA, USA), COL2 (ab34712; Abcam), MMP13 (ab39012; Abcam), MMP3 (ab53015; Abcam), ADAMTS4 (ab84792; Abcam), SPRY1 (ab111523; Abcam) and β-actin (ab8227; Abcam) for 16 h at 4 °C. Next, the membranes containing proteins were probed with the goat anti-rabbit secondary antibody (ab205718; Abcam) for 1.5 h at room temperature. Finally, protein blot signals were visualized using the enhanced chemiluminescence (ECL) detection kit (Absin).

### Bioinformatics prediction

Bioinformatics tools, including starbase3.0 (http://starbase.sysu.edu.cn/) and DianaTools (http://diana.imis.athena-innovation.gr/DianaTools/index.php?r=microT_CDS/), were used to predict the target relationship between miR-23b and circ_0020093 or SPRY1.

### Dual-luciferase reporter assay

The wild-type (WT) sequence fragment of circ_0020093 harboring miR-23b binding sites was mutated at binding sites to generate mutant sequence fragment. Similarly, the WT sequence fragment of SPRY1 3′UTR was also mutated at miR-23b binding sites to generate mutant sequence fragment. The single WT or MUT sequence fragment was cloned into the PGL4 reporter plasmid, and the fusion plasmid was named as circ_0020093-WT, circ_0020093-MUT, SPRY1-WT or SPRY1-MUT. Chondrocytes were transfected with circ_0020093-WT, circ_0020093-MUT, SPRY1-WT or SPRY1-MUT and miR-23b or miR-con and cultured for continuing 48 h. The luciferase activity was ascertained using the dual-luciferase assay system (Promega, Madison, WI, USA).

### Pull-down assay

WT sequence of miR-23b and MUT sequence of miR-23b were synthesized to prepare specific biotin-labeled probe, naming as Bio-miR-23b-WT and Bio-miR-23b-MUT, with Bio-con as a negative control. All probes were provided by Ribobio. Chondrocytes were transfected with Bio-miR-23b-WT, Bio-miR-23b-MUT or Bio-con and subsequently lysed using lysis buffer (Invitrogen). Cell lysates were incubated with streptavidin-dyna beads (Invitrogen). The RNA complexes bound on the beads were extracted with RNeasy Mini Kit (QIAGEN, Duesseldorf, Germany), and qRT-PCR assay was performed as mentioned above.

### Statistical analysis

GraphPad Prism v5.01 (GraphPad Software, La Jolla, CA) was applied for data processing. All data from at least three independent biological replications were presented as the mean ± standard deviation (SD). Difference analysis was performed using the Student’s *t*-test between two groups or using analysis of variance (ANOVA) among over two groups. Differences were deemed as statistically significant when *P*-value < 0.05.

## Result

### The expression of circ_0020093 was decreased, while the expression of miR-23b was increased in IL-1β-treated chondrocytes

**Fig. 1:**
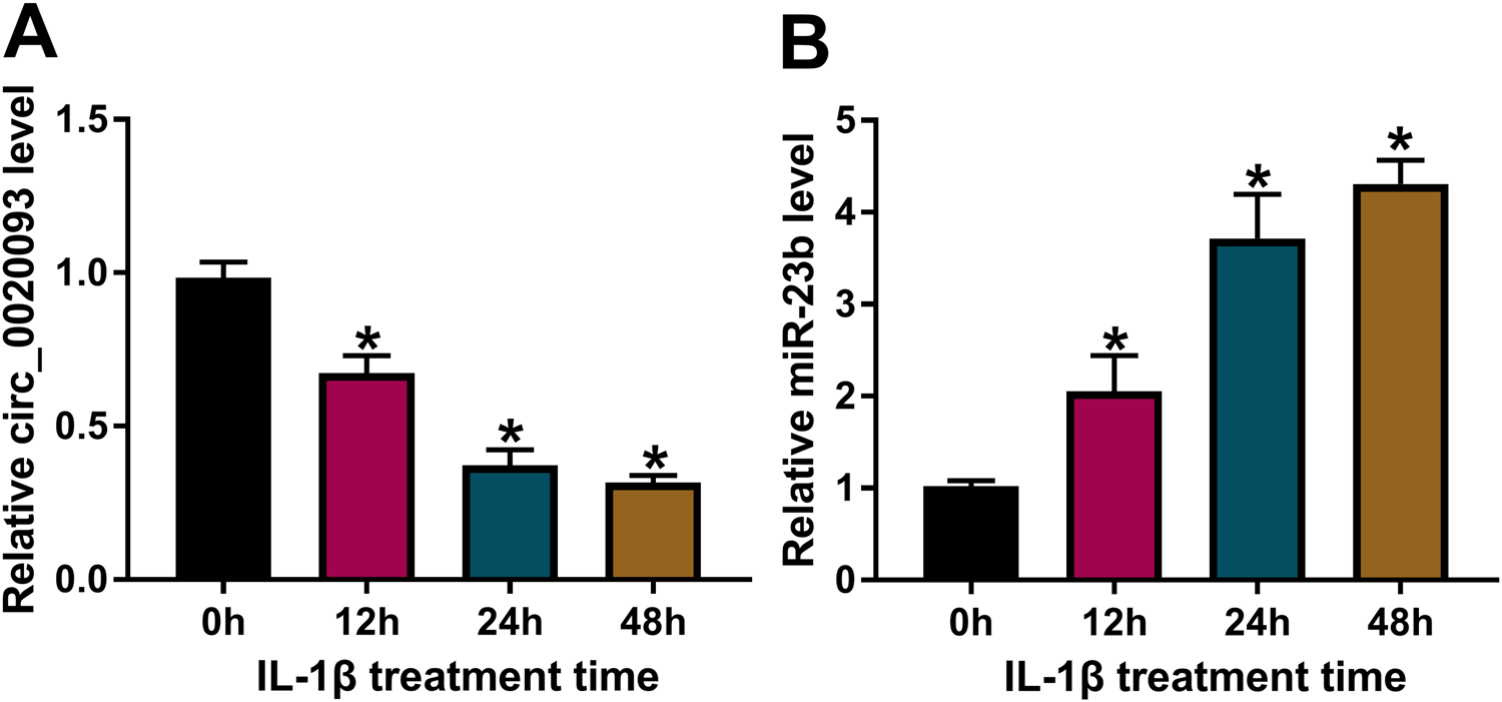
The expression of circ_0020093 was declined, while miR-23b expression was elevated in IL-1β-treated chondrocytes. In IL-1β-treated chondrocytes, **A** circ_0020093 expression and **B** miR-23b expression were detected by qRT-PCR at 0, 12, 24, and 48 h post treatment. *P < 0.05

Firstly, we examined the expression pattern of circ_0020093 and its putative target miRNA, miR-23b, in IL-1β-treated chondrocytes using qRT-PCR. The data clearly showed that circ_0020093 expression was notably declined in IL-1β-treated chondrocytes in a time-dependent manner (Fig. 1A), while miR-23b expression was elevated in IL-1β-treated chondrocytes also in a time-dependent manner (Fig. 1B). These data indicated that the dysregulation of circ_0020093 and miR-23b in IL-1β-treated chondrocytes might be associated with cartilage lesions.

### Circ_0020093 overexpression or miR-23b inhibition suppressed IL-1β-induced apoptosis and ECM degradation in chondrocytes

**Fig. 2:**
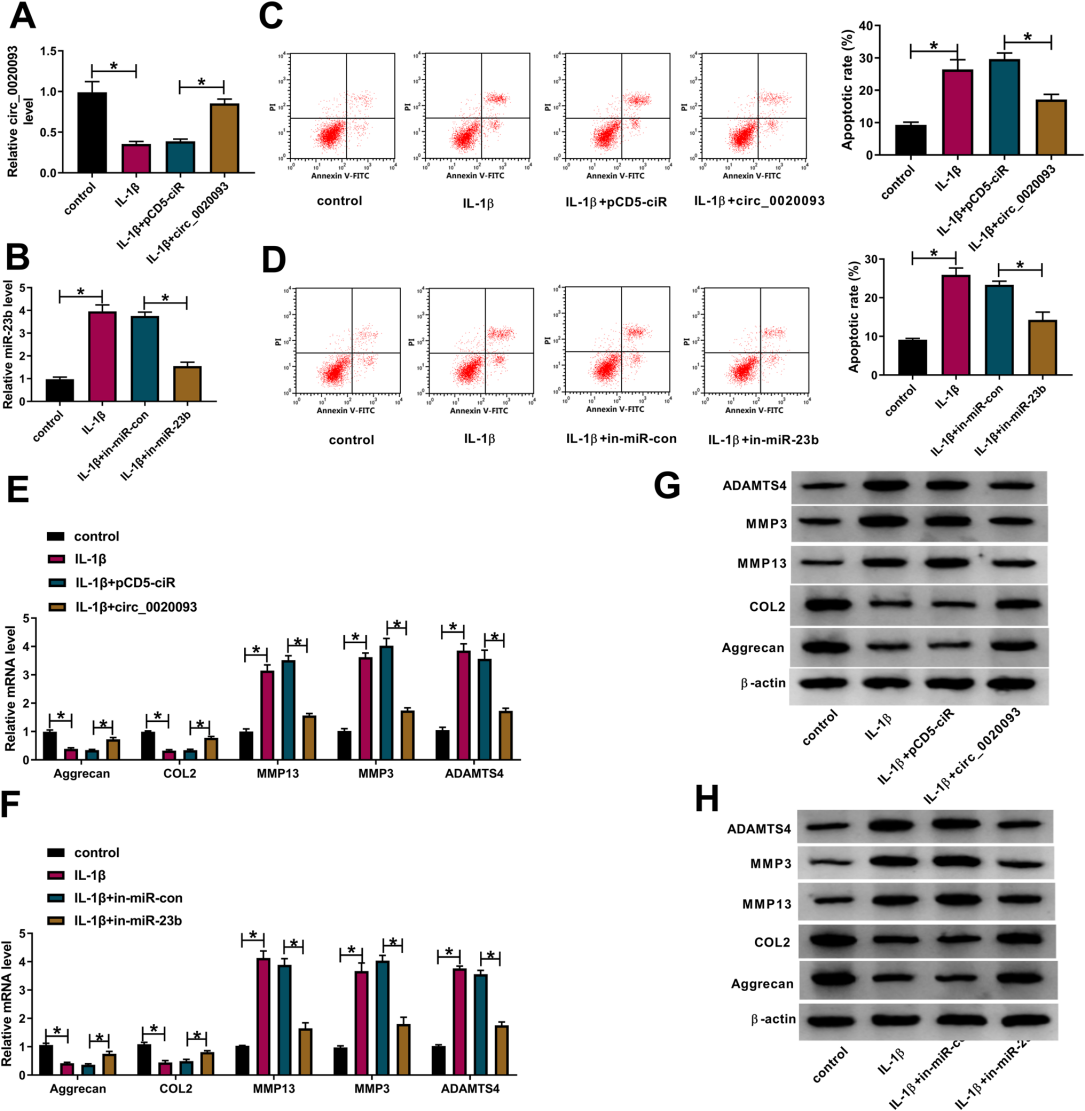
Circ_0020093 overexpression or miR-23b inhibition ameliorated IL-1β-induced chondrocyte apoptosis and ECM degradation. **A**, **B** The efficiency of circ_0020093 overexpression and miR-23b inhibition in IL-1β-treated chondrocytes was checked by qRT-PCR. **C**, **D** The effects of circ_0020093 overexpression or miR-23b inhibition on cell apoptosis were determined by flow cytometry assay. The effects of circ_0020093 overexpression or miR-23b inhibition on the expression of Aggrecan, COL2, MMP13, MMP3, and ADAMTS4 were assessed by **E**, **F** qRT-PCR and **G**, **H** Western blot. *P < 0.05

Next, the potential role of circ_0020093 and miR-23b was explored. Given that the expression of circ_0020093 was decreased in IL-1β-treated chondrocytes, we constructed overexpression plasmid to enrich the endogenous level of circ_0020093. The data showed that the expression of circ_0020093 was impaired in IL-1β-treated chondrocytes but largely recovered in IL-1β-treated chondrocytes transfected with circ_0020093 compared to pCD5-ciR (Fig. 2A), suggesting that circ_0020093 overexpression was efficient. Similarly, miR-23b expression was strikingly reduced in IL-1β-treated chondrocytes transfected with in-miR-23b (Fig. 2B), suggesting that miR-23b inhibitor was also efficient. For functional analyses, we found that the apoptotic rate in IL-1β-treated chondrocytes was significantly higher than control, while the reintroduction of circ_0020093 or in-miR-23b notably inhibited cell apoptosis (Fig. 2C, D). Moreover, ECM-related markers were quantified by qRT-PCR and western blot. The data showed that IL-1β treatment depleted the expression of Aggrecan and COL2 but promoted the expression of MMP13, MMP3 and ADAMTS4, while the reintroduction of circ_0020093 or in-miR-23b enhanced the expression of Aggrecan and COL2, but weakened the expression of MMP13, MMP3 and ADAMTS4 (Fig. 2E–H). Changes in the expression patterns of these proteins suggested that IL-1β-promoted ECM degradation was ameliorated by circ_0020093 overexpression or miR-23b inhibition.

### MiR-23b was identified to be a target of circ_0020093

**Fig. 3:**
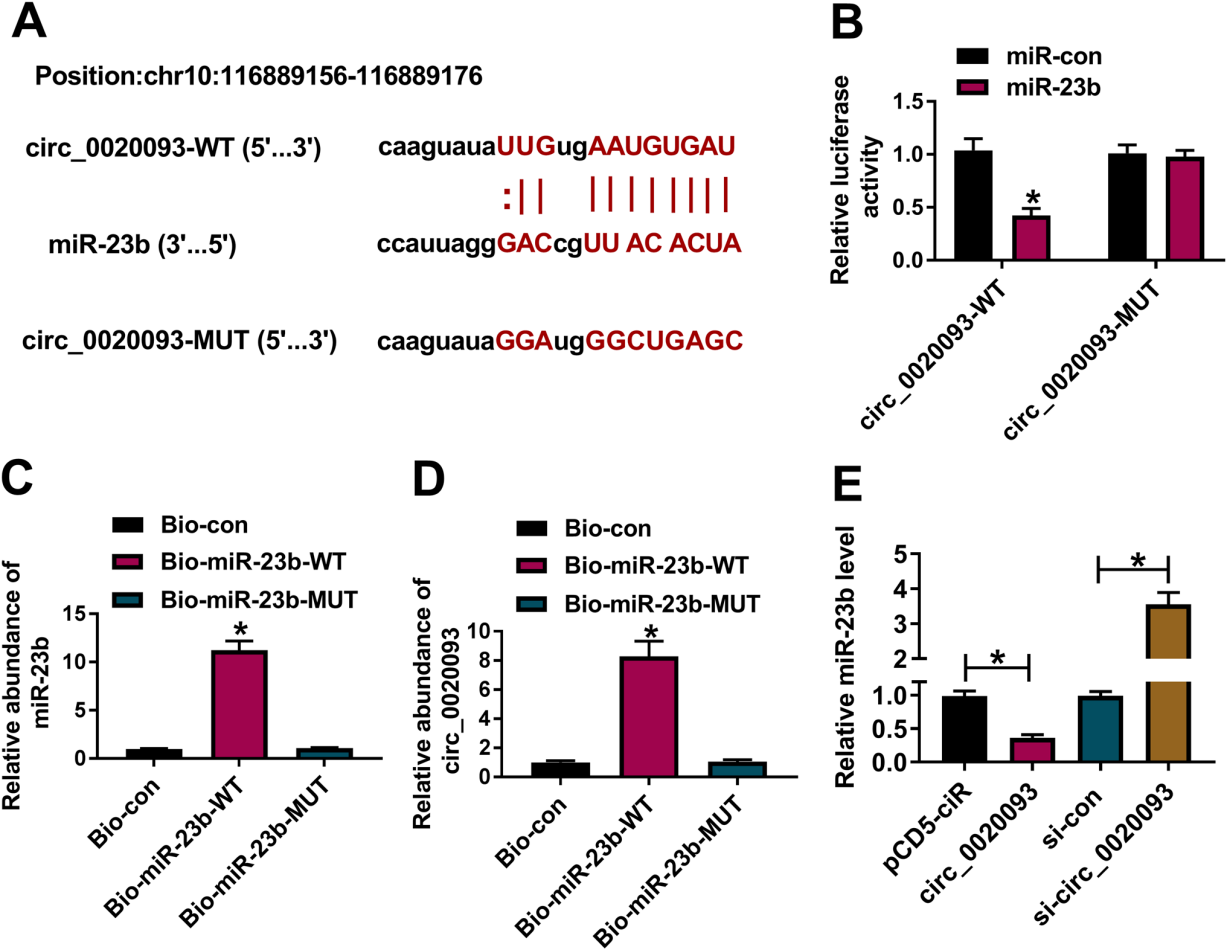
MiR-23b was a target of circ_0020093. **A** The target relationship and binding sites between circ_0020093 and miR-23b were predicted by starbase. The relationship between circ_0020093 and miR-23b was verified by **B** dual-luciferase reporter assay and **C**, **D** pull-down assay. **E** The expression of miR-23b in chondrocytes with circ_0020093 overexpression or knockdown was detected by qRT-PCR. *P < 0.05

The above findings introduced that circ_0020093 had opposite effects to miR-23b. To validate their relationship, dual-luciferase reporter assay and pull-down assay were performed. According to the wild-type binding sites between circ_0020093 and miR-23b predicted by starbase, the mutant sequence of circ_0020093 containing mutated binding sites of miR-23b was designed (Fig. 3A). Dual-luciferase reporter assay showed that the cotransfection of miR-23b and circ_0020093-WT remarkably reduced luciferase activity (Fig. 3B). For pull-down assay, biotin-labeled miR-23b probes (WT sequence and MUT sequence) were synthesized. As expected, Bio-miR-23b-WT notably enriched the abundance of miR-23b relative to Bio-miR-23b-MUT or Bio-con (Fig. 3C), thus pulling down circ_0020093 with a high abundance (Fig. 3D). Moreover, the expression of miR-23b was significantly lessened in circ_0020093-overexpressed chondrocytes, while miR-23b expression was heightened in circ_0020093-downregulated chondrocytes (Fig. 3E). Additionally, we found that the expression of miR-23b was gradually increased in chondrocytes with the gradual decrease of circ_0020093 expression (Fig. S1A and S1B). The above evidence verified that circ_0020093 directly targeted miR-23b, and thus suppressed miR-23b expression.

### Circ_0020093 alleviated IL-1β-induced apoptosis and ECM degradation in chondrocytes by targeting miR-23b

**Fig. 4:**
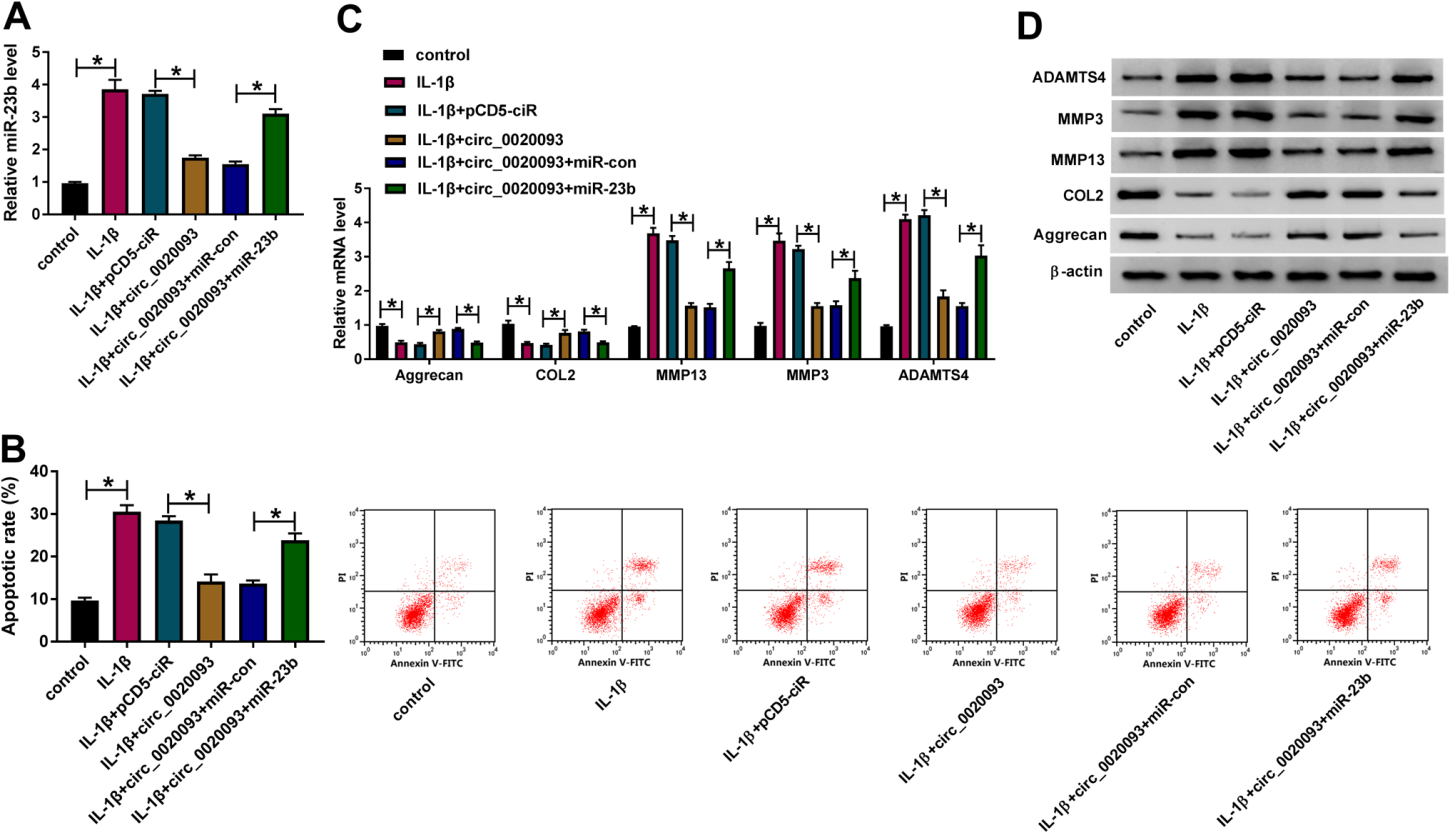
Circ_0020093 targeted miR-23b to suppress IL-1β-induced chondrocyte apoptosis and ECM degradation. IL-1β-treated chondrocytes were transfected with circ_0020093, pCD5-ciR, circ_0020093 + miR-23b, or circ_0020093 + miR-con. In these cells, **A** the expression of miR-23b was measured by qRT-PCR. **B** Cell apoptotic rate was analyzed using flow cytometry assay. **C**, **D** The expression of Aggrecan, COL2, MMP13, MMP3, and ADAMTS4 was monitored by qRT-PCR and Western blot. *P < 0.05

Rescue experiments were performed to confirm whether circ_0020093 played functions by targeting miR-23b. We transfected circ_0020093 or circ_0020093 + miR-23b into IL-1β-treated chondrocytes, with pCD5-ciR or circ_0020093 + miR-con as the corresponding control. The data from expression analysis presented that the expression of miR-23b was enhanced in IL-1β-treated chondrocytes but repressed in IL-1β-treated chondrocytes transfected with circ_0020093, while its expression was largely recovered in IL-1β-treated chondrocytes transfected with circ_0020093 + miR-23b (Fig. 4A). In function, IL-1β-induced chondrocyte apoptosis was restrained by circ_0020093 overexpression, while miR-23b reintroduction substantially promoted cell apoptosis (Fig. 4B). Besides, the expression of Aggrecan and COL2 was restored in IL-1β-treated chondrocytes transfected with circ_0020093 but largely impaired in IL-1β-treated chondrocytes transfected with circ_0020093 + miR-23b, while the expression pattern of MMP13, MMP3, and ADAMTS4 was opposite to the expression pattern of Aggrecan and COL2 in these indicated cells at both mRNA and protein levels (Fig. 4C, D). All data illustrated that circ_0020093 blocked IL-1β-induced apoptosis and ECM degradation by suppressing miR-23b expression.

### SPRY1 was a downstream target of miR-23b

**Fig. 5:**
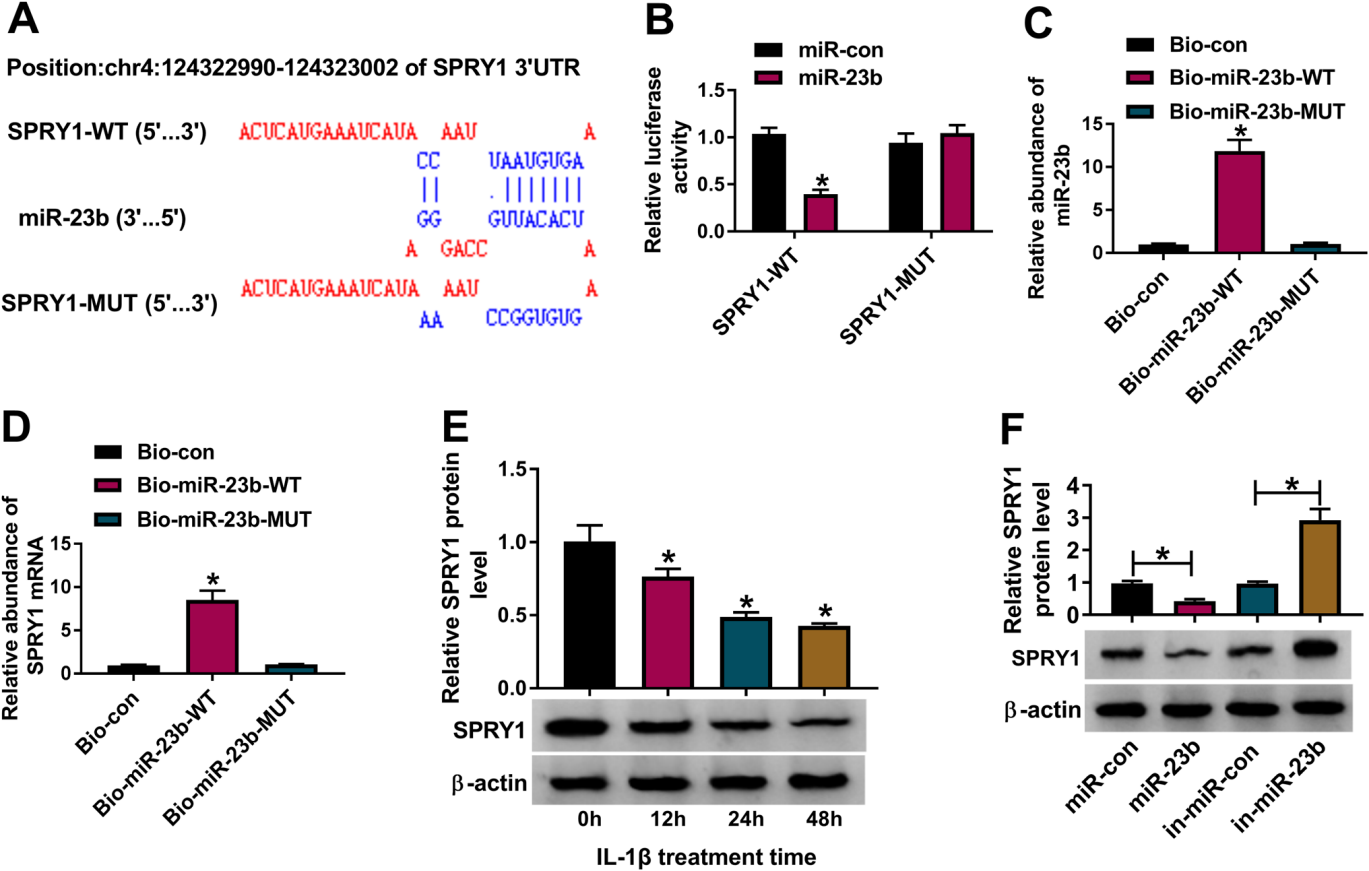
SPRY1 was a downstream target of miR-23b. **A** The relationship between SPRY1 and miR-23b was predicted by DianaTools. The interaction between miR-23b and SPRY1 was further validated by **B** dual-luciferase reporter assay and **C**, **D** pull-down assay. **E** The expression of SPRY1 at different time points post treatment of IL-1β in chondrocytes was detected by Western blot. **F** The expression of SPRY1 in chondrocytes with miR-23b overexpression or inhibition was examined using Western blot. *P < 0.05

By bioinformatics analysis of DianaTools, SPRY1 was predicted as a target of miR-23b. There were several binding sites between SPRY1 and miR-23b sequence fragments, and the mutant sequence of SPRY1 3′UTR was also generated as well as mentioned above (Fig. 5A). Dual-luciferase reporter assay showed that miR-23b reintroduction significantly diminished the luciferase activity in chondrocytes transfected with SPRY1-WT but not SPRY1-MUT (Fig. 5B). Besides, Bio-miR-23b-WT significantly enriched the abundance of miR-23b and thus combined with SPRY1 of a large abundance (Fig. 5C, D). We found the expression of SPRY1 was remarkably impaired in IL-1β-treated chondrocytes in a time-dependent manner (Fig. 5E). The expression of SPRY1 in miR-23b-overexpressed chondrocytes was declined but enhanced in miR-23b-depleted chondrocytes (Fig. 5F). In short, SPRY1 was a downstream target of miR-23b, and miR-23b could repress the expression of SPRY1.

### MiR-23b aggravated IL-1β-induced apoptosis and ECM degradation in chondrocytes by mediating SPRY1

**Fig. 6:**
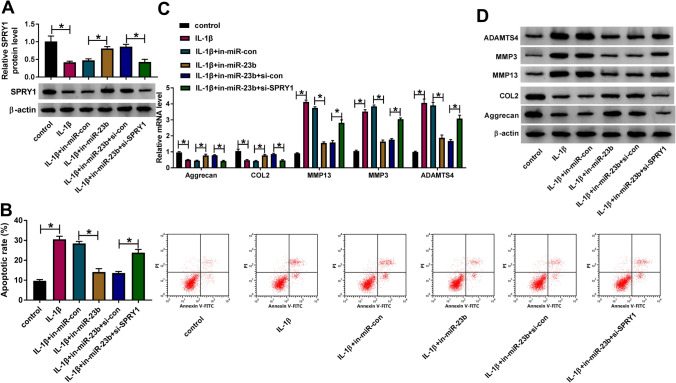
MiR-23b targeted SPRY1 to aggravate IL-1β-induced chondrocyte apoptosis and ECM degradation. IL-1β-treated chondrocytes were transfected with in-miR-23b, in-miR-con, in-miR-23b + si-SPRY1, or in-miR-23b + si-con. In these transfected cells, **A** the expression of SPRY1 was measured by Western blot. **B** Cell apoptotic rate was analyzed by flow cytometry assay. **C**, **D** The expression of Aggrecan, COL2, MMP13, MMP3, and ADAMTS4 was detected by qRT-PCR and Western blot. *P < 0.05

Subsequently, we explored whether miR-23b regulated IL-1β-induced apoptosis and ECM degradation in chondrocytes by repressing SPRY1. IL-1β-treated chondrocytes were transfected with in-miR-23b or in-miR-23b + si-SPRY1, with in-miR-con or in-miR-23b + si-con as the corresponding control. The data from Western blot showed that the expression of SPRY1 was suppressed in IL-1β-treated chondrocytes, but restored in IL-1β-treated chondrocytes transfected with in-miR-23b, while SPRY1 expression was further impaired in IL-1β-treated chondrocytes transfected in-miR-23b + si-SPRY1 (Fig. 6A). In function, IL-1β-stimulated chondrocyte apoptosis was restricted by in-miR-23b transfection but recovered by in-miR-23b + si-SPRY1 transfection (Fig. 6B). Moreover, the expression of Aggrecan and COL2 depleted by IL-1β treatment was promoted by miR-23b inhibition, while further SPRY1 knockdown repressed the expression of Aggrecan and COL2 (Fig. 6C, D). On the contrary, the expression of MMP13, MMP3 and ADAMTS4 induced by IL-1β treatment was blocked by miR-23b inhibition, while SPRY1 knockdown partly recovered their expression (Fig. 6C, D). These data suggested that SPRY1 knockdown abolished the effects of miR-23b inhibition, meaning that miR-23b aggravated IL-1β-induced apoptosis and ECM degradation in chondrocytes by mediating SPRY1.

### Circ_0020093 enriched the expression of SPRY1 by targeting miR-23b

**Fig. 7:**
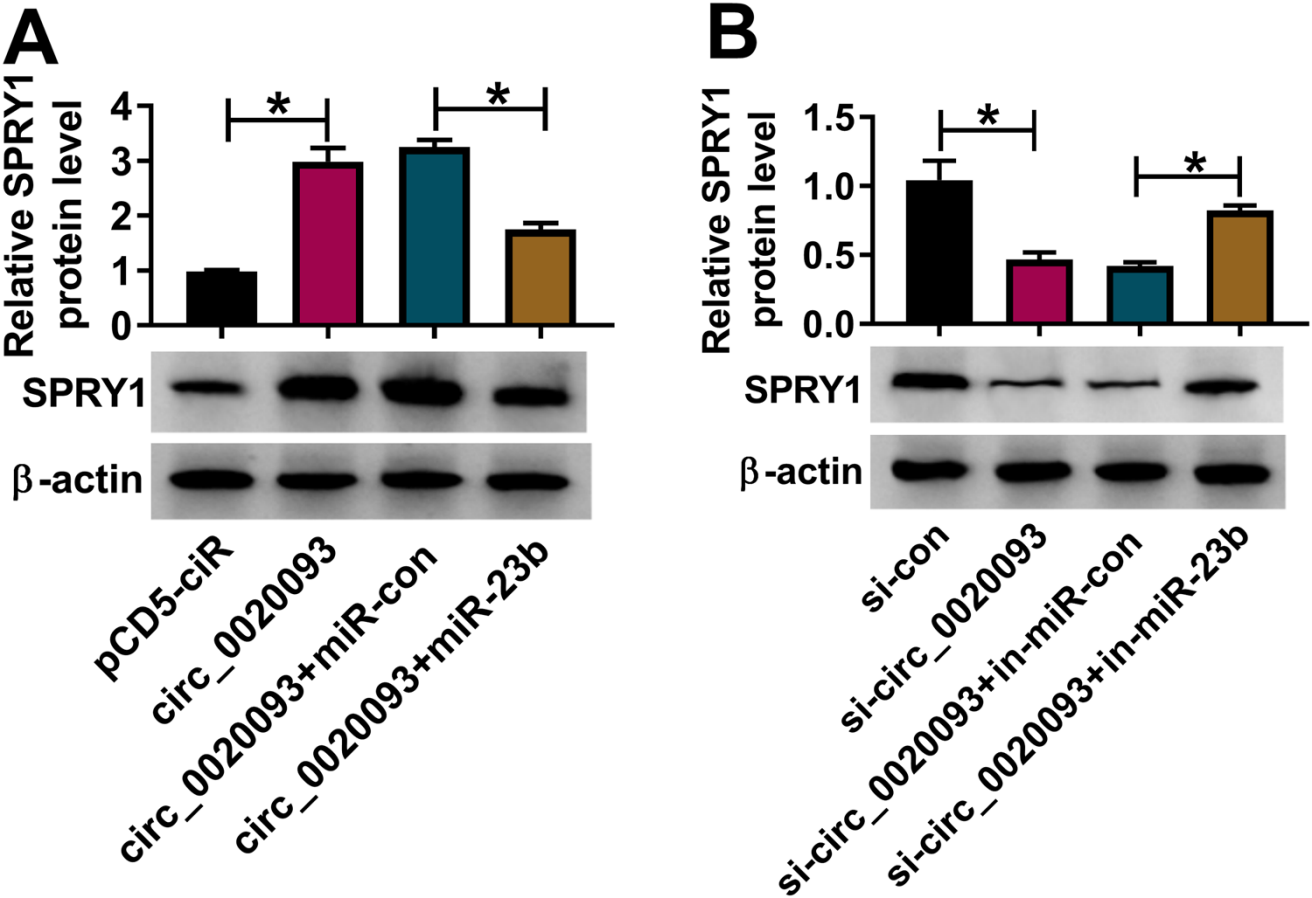
Circ_0020093 positively regulated SPRY1 expression by targeting miR-23b. **A** The expression of SPRY1 in chondrocytes transfected with circ_0020093 or circ_0020093 + miR-23b was detected by Western blot, with pCD5-ciR or circ_0020093 + miR-con as the control. **B** The expression of SPRY1 in chondrocytes transfected with si-circ_0020093 or si-circ_0020093 + in-miR-23b was detected by Western blot, with si-con or si-circ_0020093 + in-miR-con as the control. *P < 0.05

Further expression analysis demonstrated that the expression of SPRY1 was significantly promoted in chondrocytes with circ_0020093 transfection, while SPRY1 expression was lessened in chondrocytes with circ_0020093 + miR-23b transfection (Fig. 7A). In addition, the expression of SPRY1 was markedly impaired in chondrocytes transfected with si-circ_0020093 but strengthened in chondrocytes transfected with si-circ_0020093 + in-miR-23b (Fig. 7B), suggesting that circ_0020093 positively regulated SPRY1 expression by targeting miR-23b.

## Discussion

OA is a complex chronic disease with multiple pathogeneses. Though OA disturbs a large number of people worldwide [15], there are no effective strategies to prevent this disorder because the detailed mechanisms of OA have not been fully understood. Hence, more in-depth research should be conducted to clarify the development of OA. Emerging circRNAs provide novel insights to understand OA pathogenesis.

IL-1β seemed to be one of the main proinflammatory cytokines involved in the pathophysiology of OA, and its level was elevated in OA synovial fluid, synovial membrane, subchondral bone and cartilage [16]. In chondrocytes with IL-1β treatment, we found that the expression of circ_0020093 was significantly declined, which was consistent with the previous data that both IL-1β and TNF-α treatment could diminish the expression level of circ_0020093 in chondrocytes [9]. ECM degradation and chondrocyte excessive apoptosis are important features in OA [2, 5]. Aggrecan and COL2 are main components of ECM, and MMP13, MMP3 and ADAMTS4 are typical ECM-degrading enzymes in which MMP13 is a major degrading enzyme for COL2, and ADAMTS4 mainly degrades Aggrecan [17, 18]. Previous studies recorded that circ_0005105 restoration weakened the expression of Aggrecan and COL2 but strengthened the expression of MMP13 and ADAMTS4 [8], and circ_0045714 overexpression lessened the expression of CLO2 and Aggrecan [19], thereby inducing ECM degradation. In our study, we discovered that circ_0020093 reintroduction improved the expression of Aggrecan and COL2, and reduced the expression of MMP13, MMP3 and ADAMTS4 in IL-1β-treated chondrocytes, suggesting that circ_0020093 ameliorated ECM degradation. Besides, IL-1β-induced chondrocyte apoptosis was repressed by circ_0020093 overexpression. Hence, we concluded that circ_0020093 prevented IL-1β-induced chondrocyte injuries.

MiR-23b was predicted as a target of circ_0020093, which was verified by dual-luciferase reporter assay and pull-down assay. MiR-23b was documented to be upregulated in TNF-α-stimulated chondrocytes, IL-1β-induced chondrocytes, as well as OA joint tissues [12, 20]. The deficiency of miR-23b suppressed chondrocyte apoptosis and promoted the expression of COL2 and Aggrecan [20]. Besides, miR-23b restoration promoted the release of proinflammatory factors, including IL-6, IL-8 and TNF-α, and accelerated the apoptosis rate of knee joint chondrocytes [21], thus promoting the development of degenerative joint disease. In accordance with these findings, our data viewed that miR-23b inhibition blocked chondrocyte apoptosis, enhanced the expression of Aggrecan and COL2, and weakened the expression of MMP13, MMP3 and ADAMTS4, meaning that miR-23b aggravated IL-1β-induced chondrocyte injury.

Further study illustrated that miR-23b directly bound to SPRY1. The protein level of SPRY1 was markedly decreased in IL-1β-treated condylar chondrocytes [22]. Besides, SPRY1 was also demonstrated to be a target of miR-21-5p, and SPRY1 overexpression decreased the expression levels of MMP13, ACAN and ADAMTS5 that were reinforced by miR-21-5p enrichment in IL-1β-treated condylar chondrocytes [22]. In agreement with this study, the data from our study exhibited that SPRY1 expression was also decreased in IL-1β-treated chondrocytes, and SPRY1 knockdown reversed the effects of miR-23b deficiency to induce chondrocyte apoptosis and ECM degradation.

## Conclusion

In summary, our study monitored that the expression of circ_0020093 and SPRY1 was decreased, while the expression of miR-23b was enhanced in IL-1β-treated chondrocytes. Circ_0020093 upregulated SPRY1 by targeting miR-23b, thus blocking apoptosis and ECM degradation of chondrocytes. This study provided a potential therapeutic strategy for OA and proposed that circ_0020093 might prevent the progression of OA. Though the present study strongly supported the role of circ_0020093 in OA, we only elucidated these findings in cell models in vitro. These results should be reproduced in animal models in our future work.

## Supplementary Information

Below is the link to the electronic supplementary material.

Supplementary file1—Fig. S1 Circ_0020093 knockdown enhanced the expression of miR-23b. (A) The interference efficiency of si-circ_0020093#1, #2 and #3 was checked by qRT-PCR. (B) In cells transfected with si-circ_0020093#1, #2 or #3, the expression of miR-23b was detected by qRT-PCR (TIF 523 kb)

## Data Availability

The analyzed data sets generated during the present study are available from the corresponding author on reasonable request.
