# Supplementary figures and images for: Circ_0020093 ameliorates IL-1β-induced apoptosis and extracellular matrix degradation of human chondrocytes by upregulating SPRY1 via targeting miR-23b
Source: Mol Cell Biochem. 2021 May 27;476(10):3623–33. doi: 10.1007/s11010-021-04186-2 (PMC8382646; doi:10.1007/s11010-021-04186-2)

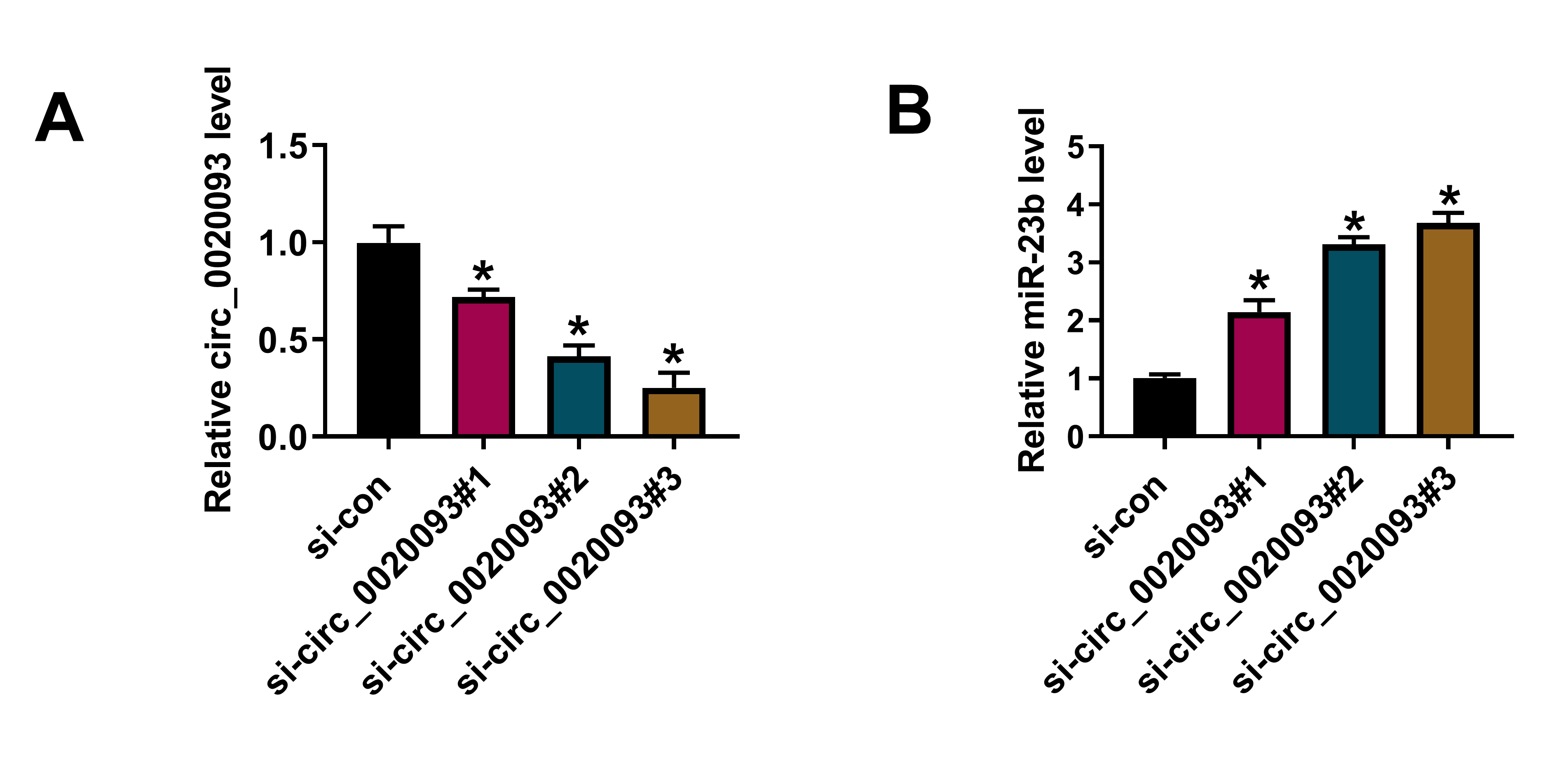

Supplement: Supplementary file 1 — Supplementary file1—Fig. S1 Circ_0020093 knockdown enhanced the expression of miR-23b. (A) The interference efficiency of si-circ_0020093#1, #2 and #3 was checked by qRT-PCR. (B) In cells transfected with si-circ_0020093#1, #2 or #3, the expression of miR-23b was detected by qRT-PCR (TIF 523 kb) [file 11010_2021_4186_MOESM1_ESM.tif]
